# Construction of a Clinical Predictive Model of Left Atrial and Left Atrial Appendage Thrombi in Patients with Nonvalvular Atrial Fibrillation

**DOI:** 10.1155/2022/7806027

**Published:** 2022-11-04

**Authors:** Lei Yin, Changjian He, Huixin Zheng, Jianshuai Ma, Jinting Liu, Xiaohong Zhang, Ruiqin Xie

**Affiliations:** Division of Cardiology, The Second Hospital of Hebei Medical University, Shijiazhuang, Hebei, China

## Abstract

**Background:**

The purpose of this study was to investigate the risk factors of left atrial (LA) or left atrial appendage (LAA) thrombi in patients with nonvalvular atrial fibrillation (NVAF) and to establish and validate relevant predictive models. It might improve thromboembolic risk stratification in patients with NVAF.

**Methods:**

This study retrospectively included 1210 consecutive patients with NVAF undergoing transesophageal echocardiography (TEE), of whom 139 patients had thrombi in LA or in LAA. Through literature review and the ten events per variable (10EPV) principle, 13 variables were finally identified for inclusion in multivariate analysis. Models were constructed by multivariate logistic stepwise regression and least absolute shrinkage and selection operator (lasso) regression.

**Results:**

After logistic regression, five variables (AF type, age, B-type natriuretic peptide, *E*/*e*' ratio, and left atrial diameter) were finally screened out as model 1. After Lasso regression, AF type, age, gender, B-type natriuretic peptide, E/e' ratio, left atrial diameter, and left ventricular ejection fraction were finally screened as model 2. After comparing the two models, the simpler model 1 was finally selected. The area under the ROC curve (AUC) of the model 1 was 0.865 (95% CI: 0.838–0.892), the Hosmer–Lemeshow test = 0.898, and the AUC = 0.861 after internal validation. The clinical decision curve showed that the new clinical prediction model could achieve a net clinical benefit when the expected threshold was between 0 and 0.6.

**Conclusion:**

This study constructed a new clinical prediction model of LA or LAA thrombi, with a higher discriminative degree than the CHADS2 and CHA2DS2-VASc scoring systems (AUC: 0.865 vs. 0.643; AUC: 0.865 vs 0.652).

## 1. Introduction

Atrial fibrillation (AF) is the most common tachyarrhythmia, characterized by irregular heart fibrillation, and is a significant risk factor for heart failure, stroke, cognitive decline, and death [1]. Among them, stroke significantly increases the morbidity and mortality of patients and increases the burden on their families and society as a whole. Therefore, the primary goal of treating patients with AF is stroke prevention. Previous studies have found that the leading cause of AF complicated by stroke is the formation and shedding of thrombus in the left atrial appendage (LAA). In patients with nonvalvular atrial fibrillation (NVAF) and stroke, up to 90% of the thrombus originates from the LAA, and the stroke caused by LAA thrombus (LAAT) has a larger embolization area and a higher fatality rate than other types [2, 3]. Left atrial spontaneous echocardiographic contrast (LASEC) and left atrial appendage sludge (LAAS) are often considered precursors of LAAT, and they also increase the risk of stroke in patients with NVAF [4–6]. LAAT, LASEC, and LAAS are collectively referred to as LA or LAA thrombi. Clinically, transesophageal echocardiography (TEE) is often performed to prevent strokes in patients with AF before cardioversion, or catheter ablation. However, it requires a high degree of patient cooperation and is not fully implemented in all centers. In addition, as a semiinvasive inspection, TEE increases patient discomfort and is not feasible in some patients (gastrointestinal bleeding and hiatus hernia) [7]. Establishing a simple and easy prediction model is necessary to screen out the high-risk groups of atrial fibrillation thrombosis and give anticoagulation therapy. This study aimed to investigate the risk factors of LAA thrombi patients with NVAF and establish and validate relevant predictive models to guide clinical treatment.

## 2. Methods

A total of 1259 patients with AF who underwent TEE examination to exclude thrombi before catheter ablation in the Second Hospital of Hebei Medical University from March 2015 to August 2021 were consecutively enrolled in this study. All patients were diagnosed with AF by ECG or 24 h Holter monitoring. The TEE was performed after low molecular heparin anticoagulant therapy.

**Figure 1:**
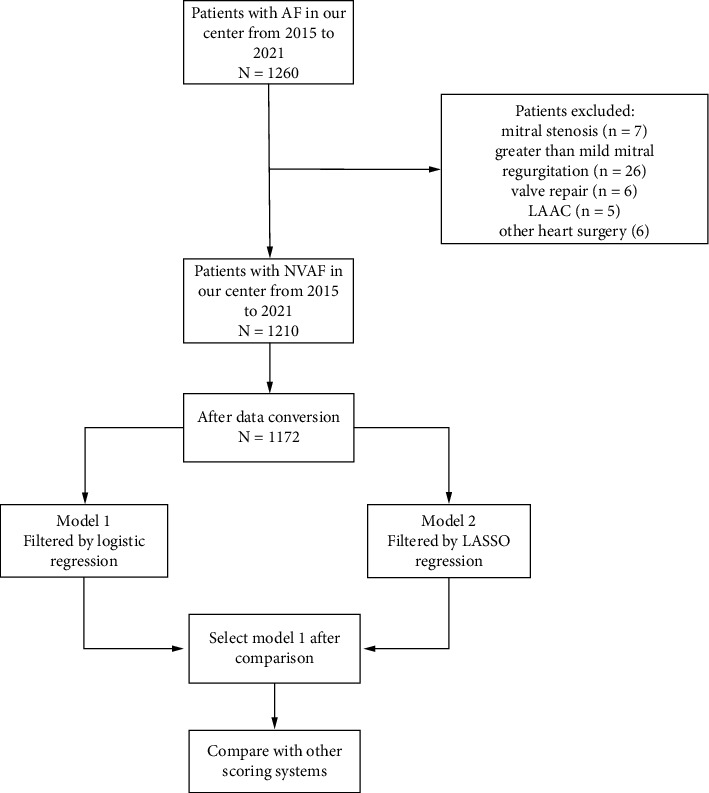
Flow chart of predictive model building.

By reviewing recently published international guidelines for the definition of NVAF and the population for oral anticoagulation [8], patients who met one of the following criteria were excluded: mitral stenosis, greater than mild mitral regurgitation, valve repair, and any artificial heart valve. Patients after left atrial appendage closure (LAAC) and the repair of congenital heart disease were also excluded. Finally, 1210 patients were enrolled in our analysis (Figure 1), including 139 with LA or LAA thrombi. Among 139 patients with thrombotic status, SEC was present in 97%, sludge was found in 13 patients, and LAAT was found in 6 patients.

The Declaration of Helsinki approved this retrospective observational study by the Ethics Committee of The Second Hospital of Hebei Medical University. Because this study was a retrospective observational research, not all participants signed an informed consent.

Baseline data such as age, gender, body mass index (BMI), smoking and drinking status, and medical history were registered. Calculate the CHADS2 score [9], and its risk factors include congestive heart failure, hypertension, age 75 years or older, diabetes mellitus, and stroke/transient ischemic attack (TIA). The calculation method is as follows: the weight of stroke/TIA is 2 points, and other factors are recorded as 1 point. At the same time, the CHA_2_DS_2_-VASc score [10] was calculated, adding vascular disease (peripheral artery disease, coronary artery disease, previous myocardial infarction, or aortic plaque), age 65–74 years, and female sex to the CHADS2 score. The calculation method is as follows: the weight of stroke/TIA/thromboembolism and age ≥ 75 years is 2 points. Congestive heart failure (clinical heart failure, objective evidence of left ventricular dysfunction, or hypertrophic cardiomyopathy), hypertension (hypertension or on antihypertensive therapy), diabetes mellitus (treatment with hypoglycaemic drugs and/or insulin or fasting blood glucose), vascular disease, age 65–74 years and female is recorded as 1 point.

Transthoracic echocardiography (TTE) was performed in all patients using a cardiac ultrasound device (iE33 system equipped with X3-1 probe; Philips Medical Systems, The Netherlands). We assessed the LAD, E-wave, e' velocity, E/e' ratio, left ventricular end-diastolic volume (LVEDV), left ventricular end-systolic volume (LVESV), and left ventricular ejection (LVEF). During the TEE examination, the ultrasound probe used is X7-2t, and experienced doctors interpret the observed images. LAAT is a well-defined, echogenic solid shadow with a border distinct from the endocardium that can be observed throughout the cardiac cycle and in all planes. LA or LAASEC is a smoke-like low echo density that can be discrete during the cardiac cycle [11]. LAAS is a dynamic, gelatinous echo density with no apparent fixed morphology but is not discrete throughout the cardiac cycle [12].

In this study, 139 patients with NVAF had thrombi, and 13 variables needed to be included according to the 10EPV principle. By consulting expert consensus, guidelines, and literature [13–20], we identified 13 prognostic factors as follows: age, gender, congestive heart failure, hypertension, diabetes mellitus, stroke/TIA, AF type, D-dimer, BNP, uric acid, *E*/*e*' ratio, LAD, and LVEF. The participants' positive incidence of vascular disease and myocardial infarction were low (<5%) and were not entered into multivariate analysis. Due to the log10 transformation of D-dimer and BNP, some data were missing, and finally, only 1172 patients were entered into the multivariate analysis.

## 3. Statistical Analysis

R software conducted all statistical analyses (https://www.r-project.org, The R Foundation). Normally distributed continuous variables are presented as the mean ± standard deviation (SD), and abnormally distributed data are presented as median (*Q*_*L*_, *Q*_*U*_). The Student's *t*-test or the rank-sum test compared the continuous variables. Categorical variables are presented as frequencies and percentages. To compare the categorical variables, the Pearson chi-square test was performed. We use single imputation with chained equations to replace missing values of BNP (24%), BMI (19%), D-dimer (19%), *E*/*e*' ratio (17%), LAD (3%), and LVEF (3%), and use these values in our primary analysis. According to the principle of 10EPV, a total of 13 variables were included in the multivariate analysis. Levels of D-dimer and BNP in the imputation model were converted to a normal distribution by log10 transformation and entered into multivariate analysis. All the continuous variables included in the model have a linear relationship with the outcome. Logistic stepwise regression and LASSO regression were used for multivariate analysis. We use the area under the curve (AUC), net reclassification index (NRI), integrated discrimination improvement (IDI), and likelihood ratio test (LRT) to verify the pros and cons of the two models. Finally, screened factors were used to construct a nomogram to predict the risk probability of LA or LAA thrombi. Internal validation of the nomogram was performed using bootstrapping with 1000 resamples. The discriminative ability of the nomogram was assessed by the receiver operating characteristics (ROC) curves and AUC. The Hosmer–Lemeshow test assessed the accuracy ability of the nomogram. A calibration plot was employed for comparing predicted results and actual outcomes. In addition, decision curve analysis (DCA) was used to evaluate the nomogram's clinical outcomes and benefits by comparing the model's threshold probabilities range to that of CHADS2 and CHA2DS2-VASc scoring systems.

## 4. Results

The baseline characteristics of the patients are presented in Table 1. Patients with thrombi were generally older, and most of them had a history of myocardial infarction, heart failure, stroke/TIA, and persistent AF. Laboratory parameters such as red cell distribution width (RDW), D-dimer, fibrinogen, BNP, creatinine, and uric acid are higher in patients with thrombi. Various cardiac function indicators in TTE are worse than those without thrombi.

Multivariate logistic analysis showed that age, AF type, BNP, *E*/*e*' ratio, and LAD were independent risk factors for thrombi. Relevant data from Model 1 are shown in Table 2. The variables screened by the multivariate logistic analysis were used to construct Model 1. The AUC of Model 1 was 0.865 (95% CI: 0.838–0.892), and the Hosmer–Lemeshow test was 0.898. LASSO regression showed that age, gender, AF type, BNP, *E*/*e*' ratio, LAD, and LVEF were independent risk factors for thrombi. The variables screened by LASSO regression are used to build Model 2. The AUC of Model 2 was 0.866 (95% CI: 0.839–0.893), and the Hosmer–Lemeshow test was 0.790. The variables of Models 1 and 2 are still linear in the multivariate model. There was no significant difference between Model 1 and Model 2 in AUC, NRI, IDI, and LRT (Table 3). However, Model 1 consists of fewer variables, and it is more conducive to clinical application. We will use Model 1 for further analysis.

**Figure 2:**
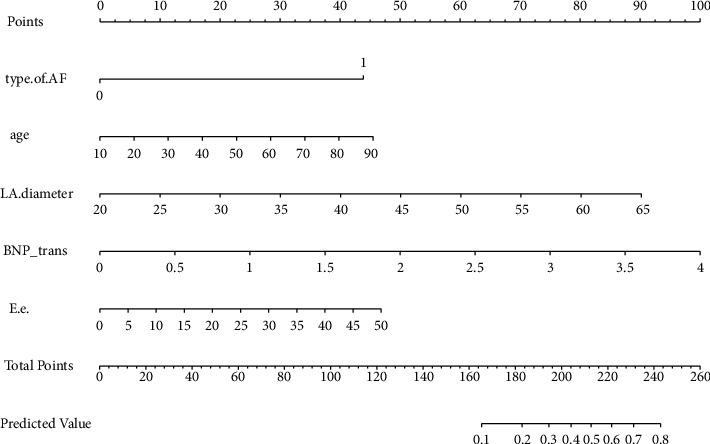
Nomogram for predicting LA/LAA thrombi in patients with NVAF. BNP_trans: The log10-transformed value of BNP. The total score is obtained by adding the scores corresponding to the variables in the nomogram. The risk value corresponding to the total score is the estimated probability of LA/LAA thrombi.

**Figure 3:**
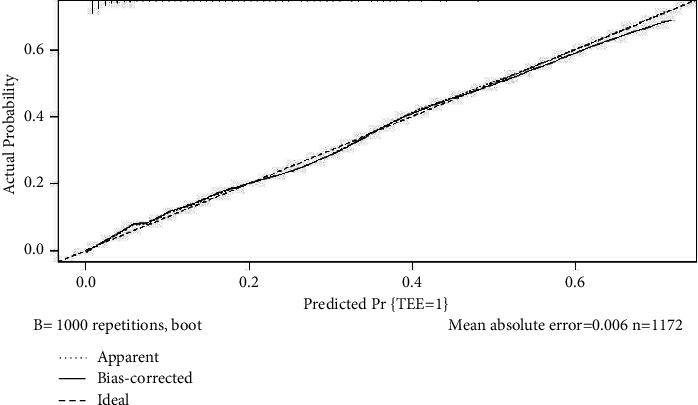
Calibration plot for predicting LA/LAA thrombi.

The nomogram was constructed to predict LA or LAA thrombi probability based on the screened factors of Model 1 (Figure 2). The web version of the nomogram is available at https://kawhi10.shinyapps.io/dynnomapp/. The C-indices obtained by the nomogram after internal verification are 0.861. Meanwhile, the calibration plot after internal verification displayed exemplary compliance between actual observations and predicted results (Figure 3).

**Figure 4:**
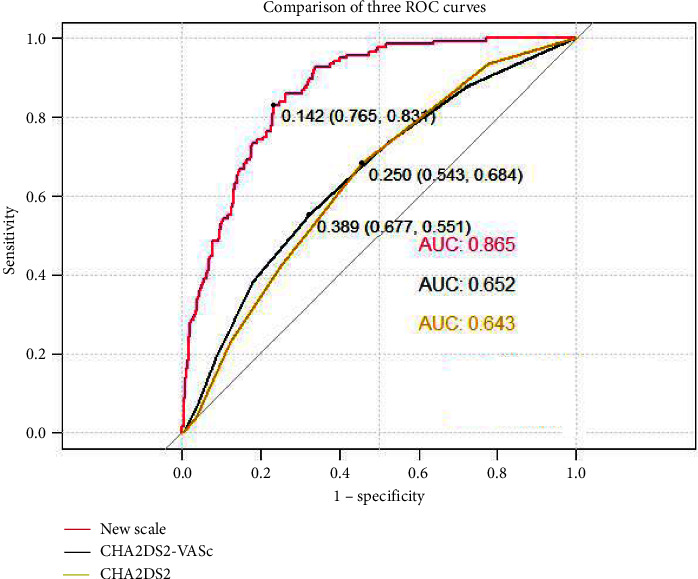
ROC curves, AUC, and an optimal cut-off value of the three scoring systems.

**Figure 5:**
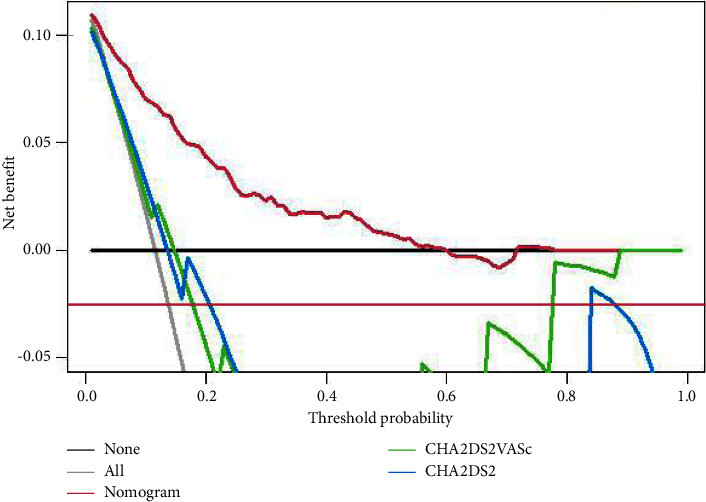
Clinical decision curve analysis of the three scoring systems.

The discriminative ability of the nomogram was better than CHADS2 and CHA2DS2-VASc scoring systems, with higher AUCs (0.861 vs. 0.643; 0.861 vs. 0.652) for thrombi (Figure 4). Besides, we compared the clinical benefits of the nomogram to that of CHADS2 and CHA2DS2-VASc scoring systems by performing DCA. As shown in Figure 5, the nomogram's DCA curves exhibited more extensive net benefits by a wide range of threshold probability than CHADS2 and CHA2DS2-VASc scoring systems, indicating that our model has better clinical performance practicality.

## 5. Discussion

Compared with the precious scoring systems for predicting thrombosis risk, this study has the following advantages: (1) This paper is strict in the selection of predictor variables. The selected predictors were clinically meaningful and readily available. (2) Our final prediction model uses relatively few variables but achieves higher AUC than other scoring systems. (3) Most of the selected variables are continuous variables, making the model's prediction ability more accurate. (4) A web-based prediction tool has been developed, which is convenient for clinicians to quickly calculate the thrombosis risk of NVAF patients and give corresponding anticoagulation or surgical treatment.

Many scores, such as the CHADS2 and CHA2DS2-VASc scoring systems, have been developed to guide physicians in starting anticoagulation. Nevertheless, the CHADS2 and CHA2DS2-VASc scoring systems mainly evaluate patients' stroke risk with NVAF. The ability of these two scoring systems to predict thrombotic status is controversial [14, 15, 21]. Our study showed that the discrimination of CHADS2 score and CHA2DS2-VASc score in predicting thrombi were 0.643 (95% CI: 0.598–0.688) and 0.652 (95% CI: 0.603–0.700), and the risk prediction with these models is poor. Chen et al. [22]. constructed a model for predicting LAAT based on three independent risk factors: left atrial appendage emptying velocity (LAAEV), LA or LAASEC, and less than moderate to severe mitral regurgitation [AUC = 0.88, 95% CI: 0.82–0.95]. LAAEV [22, 23] and LAA morphology [24] are often considered risk factors for LAAT, but considering that they are mainly measured by TEE, they are not included in multivariate analysis. Due to the different medical levels of center levels, we tried to choose simple and easily available indicators for further research. Han et al. [14] and Cai et al. [25] entered the estimated glomerular filtration rate (GFR) < 60 ml/min/1.73 m^2^ into the scoring systems for predicting the presence of LAT/SEC. The estimated GFR was evaluated by age, sex, creatinine, and race [26]. But this formula, for evaluating, GFR is only suitable for patients with chronic kidney disease, and if applied to normal people, the true GFR will be underestimated [26]. In our study population, only 33 patients were diagnosed with renal dysfunction, and most of the patients had a normal renal function. Fu et al. [15]. established the scoring system of LA or LAA thrombi through six classification variables such as NT-proBNP, blood type A, LAD, age, previous HF, and previous stroke/TIA. Cut-off values for categorical variables were obtained from the ROC curve. However, the author does not evaluate the model's accuracy, and it is relatively simple to deal with the weight of each variable.

The confounding factor of oral anticoagulants was not included in this study. However, almost all patients included in our center were first-diagnosed AF patients, and the proportion of oral anticoagulants was deficient. Based on a recent meta-analysis of left atrial appendage thrombi [27], we screened out studies that did not give oral anticoagulants before TEE. The incidence of LAAT in the selected studies ranged from 0% to 9.6% (Table S1), which is basically in line with our incidence of LAAT. Schaeffer et al. [28] had a higher incidence of LAAT (9.6%), which may be related to the lack of anticoagulation. The prevalence rate of sludge and SEC was 1.0% and 9.7% in patients undergoing catheter ablation according to the recent meta-analysis [27], and basically the same rate as ours.

AF type, LAD, age, BNP, and *E*/*e*' ratio were independent risk factors for predicting LA or LAA thrombi in this study. AF has been identified in recent studies as a marker for the progression of atrial cardiomyopathy [29, 30]. Incorporating the type of AF into the prediction model can better evaluate the inflammatory response of the atrial myocardium, thereby improving the accuracy of predicting thrombosis status. Left atrial size is closely related to AF, and persistent AF usually causes atrial enlargement, promoting thrombus formation. Due to the lack of data on left atrial volume, we used left atrial diameter instead of left atrial size to enter the analysis [31]. Our results show that both left atrial diameter and atrial fibrillation type are independent risk factors for thrombotic status, which may suggest that the left atrial thrombi does not necessarily require atrial fibrillation and may occur in the context of other manifestations of the atrial disease [29, 30, 32]. Age was included in the CHADS2 score and CHA2DS2-VASc score as a risk factor for stroke [9, 13]. We included age as a variable in multivariate analysis and proved that age was also an independent risk factor for thrombi. This may be related to the decrease in LAAEV rate with increasing age in AF patients [33]. Elevations of BNP and NT-proBNP are generally associated with atrial and ventricular dysfunction. Kamel et al. [32] found a strong association between elevated NT-proBNP and stroke even after excluding AF and heart failure, suggesting that NT-proBNP may reflect atrial or other pathways associated with thromboembolism. Our study indicates that BNP may cause stroke through the atrial thrombus pathway. The *E*/*e*' ratio is a sign of left ventricular diastolic function. Ishikawa et al. [20] showed that the increased E/e' ratio was robustly associated with the presence of silent brain infarction independent of the CHA2DS2-VASc score.

After completing the construction of the model, this study also tested the differentiation, accuracy, and clinical net benefit of the model. The AUC estimates the probability that the predicted results of the model are consistent with the actual observed results. The model AUC and internal verification AUC established in this study are all more than 85%, indicating that the model has a good guiding significance for predicting the incidence of LA or LAA thrombi in patients with NVAF. The accuracy reflects the consistency between the predicted risk and the actual risk. The Hosmer–Lemeshow test shows that the model's prediction fits well with the actual situation. Clinical decision curves are often used to evaluate the net benefits of predictive models in clinical use. This prediction threshold is 0–0.6. However, many randomized controlled studies still need to verify the determination of a specific probability threshold.

### 5.1. Limitation

1. This study is a single-center retrospective study, and the identified risk factors are highly correlated with the characteristics of the patients admitted. In addition, only internal validation was performed in this study, and external validation with data from other centers is still needed to be verified for the reproducibility of this clinical prediction model. 2. Although the CHA2DS2-VASc score is calculated according to the latest guidelines, some score indicators are not routinely screened (e.g., aortic plaque and coronary angiography), which may underestimate the score. 3. The clinical data of the included study population were missing at the time of entry. This study did not adopt the multiple imputation method to deal with the missing values but adopted the single imputation method. This may cause data offset to some extent.

## 6. Conclusion

The study constructed a new clinical prediction model of LA or LAA thrombi in patients with NVAF, which showed good performance in terms of discrimination, accuracy, and net clinical benefit. However, the external performance of this predictive model still needs to be verified by multicenter clinical data.

## Figures and Tables

**Table 1:** Comparison of the baseline condition between non-LA/LAA thrombi and LA/LAA thrombi group.

Variables	Overall	LA/LAA thrombi (-)	LA/LAA thrombi (+)	*P* Value
*n*	1210	1071	139	
Age, years (mean ± SD)	61.20 (10.08)	60.80 (10.12)	64.29 (9.29)	<0.001
Male, *n* (%)	720 (59.5)	645 (60.2)	75 (54.0)	0.185
BMI, kg/m^2^ (mean ± SD)	26.75 (12.89)	26.65 (11.76)	27.53 (19.57)	0.448
Smoke, *n* (%)	231 (19.1)	211 (19.7)	20 (14.4)	0.166
Alcohol, *n* (%)	204 (16.9)	180 (16.8)	24 (17.3)	0.987
Hypertension, *n* (%)	683 (56.4)	595 (55.6)	88 (63.3)	0.1
DM, *n* (%)	204 (16.9)	175 (16.3)	29 (20.9)	0.223
Myocardial infarction, *n* (%)	30 (2.5)	23 (2.1)	7 (5.0)	0.077
Previous HF, *n* (%)	500 (41.3)	398 (37.2)	102 (73.4)	<0.001
Vascular disease, *n* (%)	46 (3.8)	39 (3.6)	7 (5.0)	0.567
Stroke/TIA, *n* (%)	301 (24.9)	257 (24.0)	44 (31.7)	0.063
Coronary heart disease, *n* (%)	370 (30.6)	320 (29.9)	50 (36.0)	0.171
Paroxysmal AF, *n* (%)	698(57.7)	686(64.1)	12(8.6)	<0.001
Persistent AF, *n* (%)	512 (42.3)	385 (35.9)	127 (91.4)	<0.001
CHA2DS2 score, (M [*Q*_L_, *Q*_U_])	1.00 [1.00, 3.00]	1.00 [1.00, 3.00]	2.00 [1.00, 3.00]	<0.001
CHA2DS2-VASc score, (M [*Q*_L_, *Q*_U_])	3.00 [1.00, 4.00]	3.00 [1.00, 4.00]	4.00 [2.00, 5.00]	<0.001
RDW, % (mean ± SD)	42.41 (3.87)	42.27 (3.90)	43.52 (3.44)	<0.001
LDL-C, mmol/L (mean ± SD)	2.52 (0.79)	2.52 (0.79)	2.52 (0.79)	0.941
D-dimer, *μ*g/L (M [*Q*_L_, *Q*_U_])	0.08 [0.05, 0.15]	0.08 [0.05, 0.15]	0.10 [0.06, 0.19]	0.003
Fib, g/L (mean ± SD)	2.91 (1.06)	2.88 (1.05)	3.18 (1.11)	0.002
BNP, pg/mL (M [*Q*_L_, *Q*_U_])	87.45 [37.00, 202.75]	73.30 [31.80, 178.50]	251.00 [130.50, 413.50]	<0.001
Cr, mg/dL (mean ± SD)	72.72 (21.16)	72.21 (21.42)	76.63 (18.72)	0.021
Uric acid, *μ*mol/L (mean ± SD)	324.13 (93.21)	321.02 (91.96)	348.15 (99.47)	0.001
E-wave, cm/s (mean ± SD)	80.93 (32.31)	78.67 (31.47)	98.42 (33.44)	<0.001
e' velocity, cm/s (mean ± SD)	6.41 (2.01)	6.45 (2.00)	6.12 (2.02)	0.073
E/e' ratio (mean ± SD)	13.38 (5.31)	12.88 (4.87)	17.25 (6.78)	<0.001
LA diameter, mm (mean ± SD)	37.90 (5.66)	37.24 (5.35)	42.99 (5.41)	<0.001
LVEDV, mL (mean ± SD)	102.45 (26.83)	101.78 (26.17)	107.62 (31.08)	0.016
LVESV, mL (mean ± SD)	39.96 (15.73)	39.03 (14.91)	47.12 (19.64)	<0.001
LVEF, % (mean ± SD)	61.44 (7.69)	61.99 (7.26)	57.17 (9.39)	<0.001

Abbreviations: BMI, body mass index; DM, diabetes mellitus; HF, heart failure; RDW, red cell distribution width; LDL –C, low-density lipoprotein; BNP, B-type natriuretic peptide; Cr, creatinine; LA, left atrial; LVEDV, left ventricular end-diastolic volume; LVESV, left ventricular end-systolic volume; LVEF, Left ventricular ejection fraction.

**Table 2:** Predictors of LA/LAA thrombi screened by multivariate logistic regression.

Predictors	Estimated *β*	OR	95% Cl	*p* value
Persistent AF	2.029	7.6	4–14.46	<0.001
Age	0.026	1.03	1–1.05	0.025
LAD	0.093	1.1	1.05–1.14	<0.001
BNP	1.156	3.18	1.89–5.34	<0.001
E/e' ratio	0.043	1.04	1.01–1.08	0.021

Abbreviations: AF, atrial fibrillation; LAD, left atrial diameter; BNP, B-type natriuretic peptide; OR, odds ratio; CI, confidence interval.

**Table 3:** Comparison between models 1 and 2.

	AUC	NRI (categorical)	NRI (continuous)	IDI	LRT
*p* value	0.953	0.145	0.565	0.204	0.502

## Data Availability

The experimental data used to support the findings of this study are available from the corresponding author upon request.

## Supplementary Materials

Supplementary Materials Supplementary material associated with this article can be found.
