# Supplementary material for: Construction of a Clinical Predictive Model of Left Atrial and Left Atrial Appendage Thrombi in Patients with Nonvalvular Atrial Fibrillation
Source: J Interv Cardiol. 2022 Nov 4;2022:7806027. doi: 10.1155/2022/7806027 (PMC9652078; doi:10.1155/2022/7806027)
Supplement: Supplementary Materials — Supplementary material associated with this article can be found. [file 7806027.f1.docx]

| Author | Year of publication | Country | Sampling | Inclusion criteria | Exclusion criteria | Treatment | Sample (n) | case (n) |
| --- | --- | --- | --- | --- | --- | --- | --- | --- |
| Roijer^[1]^ | 2000 | Sweden | Consecutive | Non-rheumatic AF or AFL, lasting >48 h | NR | Low molecular weight heparin (dalteparin) , or no therapy | 242 | 14 |
| Schaeffer^[2]^ | 2018 | Germany | Consecutive | Haemodynamically stable, nonreversible cause of AF, and AF lasting ≥48 hours | NR | No therapy | 136 | 13 |
| Milhem^[3]^ | 2019 | France | Consecutive | Patients admitted for catheter ablation of AF | NR | NR | 235 | 1 |
| Anselmino^[4]^ | 2017 | Italy | Consecutive | Patients undergoing pre-procedural TEE before (> 48 hours) elective AF transcatheter ablation | The presence of hypertrophic cardiomyopathy (HCM),dilated cardiomyopathy (DCM), restrictive cardiomyopathy (RCM), constrictive pericarditis and valvular cardiomyopathy, defined as valvular defect of at least moderate grade, previous heart surgery and a left ventricle ejection fraction (EF) ≤ 35%. | A minimum of a 5 day-course of low molecular weight heparin at weight adjusted dose (80-100U/Kg every 12 hours) was prescribed. | 950 | 2 |
| Gunawardene^[5]^ | 2017 | Germany | Consecutive | Patients scheduled for catheter ablation of AF | Patients with device thrombus or mechanical valve thrombosis were excluded | Received no OAC prior to ablation or heparin bridging (with either low molecular weight heparin or unfractionated heparin) | 243 | 0 |
| Cresti^[6]^ | 2019 | Italy | Consecutive | Patients with paroxysmal or persistent atrial tachyarrhythmias, candidates to cardioversion, who opted for a transoesophageal echocardiography-guided strategy | Acute coronary syndrome, acute pulmonary embolism, acute stroke or transient ischaemic attack, history of rheumatic valve disease, mitral stenosis, mechanical prosthesis. | Heparin, Antiplatelet, or no therapy | 642 | 51 |

Supplementary Table 1. Prevalence of thrombus in patients not on oral anticoagulation

ECV: electrical cardioversion; CA: catheter ablation; NR: not reported.

**References**

[1] Roijer A, Eskilsson J, Olsson B. Transoesophageal echocardiography-guided cardioversion of atrial fibrillation or flutter. Selection of a low-risk group for immediate cardioversion. Eur Heart J. 2000. 21(10): 837-47.

[2] Schaeffer B, Rüden L, Salzbrunn T, et al. Incidence of intracardiac thrombus formation prior to electrical cardioversion in respect to the mode of oral anticoagulation. J Cardiovasc Electrophysiol. 2018. 29(4): 537-547.

[3] Milhem A, Ingrand P, Tréguer F, et al. Exclusion of Intra-Atrial Thrombus Diagnosis Using D-Dimer Assay Before Catheter Ablation of Atrial Fibrillation. JACC Clin Electrophysiol. 2019. 5(2): 223-230.

[4] Anselmino M, Garberoglio L, Gili S, et al. Left atrial appendage thrombi relate to easily accessible clinical parameters in patients undergoing atrial fibrillation transcatheter ablation: A multicenter study. Int J Cardiol. 2017. 241: 218-222.

[5] Gunawardene MA, Dickow J, Schaeffer BN, et al. Risk stratification of patients with left atrial appendage thrombus prior to catheter ablation of atrial fibrillation: An approach towards an individualized use of transesophageal echocardiography. J Cardiovasc Electrophysiol. 2017. 28(10): 1127-1136.

[6] Cresti A, García-Fernández MA, Sievert H, et al. Prevalence of extra-appendage thrombosis in non-valvular atrial fibrillation and atrial flutter in patients undergoing cardioversion: a large transoesophageal echo study. EuroIntervention. 2019. 15(3): e225-e230.
